# Next-Generation DNA Sequencing of Grade 1 Meningioma Tumours: A Case Report of Angiomatous and Psammomatous Meningiomas

**DOI:** 10.7759/cureus.54009

**Published:** 2024-02-11

**Authors:** Mohiuddin M Taher, Khalid M Ashour, Bashayer A Althaqafi, Albatool Mansouri, Arwa A Al-Harbi, Weam Filfilan, Ghassan Y Bakhsh, Najwa A Bantan, Muhammad Saeed, Khalid AlQuthami

**Affiliations:** 1 Science and Technology Unit and Deanship of Scientific Research, Umm Al-Qura University, Makkah, SAU; 2 Medical Genetics, Umm Al-Qura University, Makkah, SAU; 3 Neurological Surgery, Alexandria University, Alexandria, EGY; 4 Neurosurgery, Al-Noor Specialty Hospital, Ministry of Health, Makkah, SAU; 5 Neurosurgey, Al-Noor Specialty Hospital, Ministry of Health, Makkah, SAU; 6 Pathology and Laboratory Medicine, Al-Noor Specialty Hospital, Ministry of Health, Makkah, SAU; 7 General Medicine, King Saud Bin Abdulaziz University for Health Sciences College of Medicine, Makkah, SAU; 8 Radiology, Al-Noor Specialty Hospital, Ministry of Health, Makkah, SAU; 9 Laboratory Medicine and Blood Bank, Al-Noor Specialty Hospital, Ministry of Health, Makkah, SAU

**Keywords:** genetic variants, gene mutations, low grade gliomas, ion proton, saudi arabia, brain cancer, psammomatous meningioma, angiomatous meningioma, low-grade meningiomas, next generation dna sequencing

## Abstract

We performed the next-generation sequencing (NGS) analysis of a rare grade 1 brain meningioma (angiomatous type) and a common grade 1 spinal meningioma (psammomatous type) and compared their mutation profiling. The data were analysed using the Ion Reporter 5.16 programme (Thermo Fisher Scientific, Waltham, MA). Sequencing analysis identified 10 novel variants and two previously reported variants that were common between these two tumours. Nine variants were missense, which included an insertion in EGFR c.1819_1820insCA, causing frameshifting, and a single nucleotide deletion in HRAS and HNF1A genes, causing frameshifting in these genes. These were common variants identified for both tumours. Also, 10 synonymous variants and 10 intronic variants were common between these two tumours. In intronic variants, two were splice site_5’ variants (acceptor site variants). Typical of the angiomatous type tumour, there were 11 novel and six previously reported variants that were not found in the psammomatous tumour; three variants were synonymous, 11 were missense mutations, and three were deletions causing frameshifting. The deletion variants were in the SMARCB1, CDH1, and KDR genes. In contrast, eight novel and five previously reported variants were found in the psammomatous meningioma tumour. In this tumour, two variants were synonymous: a deletion causing a frameshifting in [(c.3920delT; p. (Ile1307fs)], and a two-base pair insertion and deletion (INDEL) [(c.3986_3987delACinsGT; p. (His1329Arg)] both in the APC gene were also found. Among our findings, we have identified that ALK, VHL, CTNNB1, EGFR, ERBB4, PDGFRA, KDR, SMO, ABL1, HRAS, ATM, HNF1A, FLT3, and RB1 mutations are common for psammomatous meningioma and angiomatous tumours. Variants typical for angiomatous (brain) meningioma are PIK3CA, KIT, PTPN11, CDH1, SMAD4, and SMARCB1; the variants typical for psammomatous meningioma are APC, FGFR2, HNF1A, STK11, and JAK3. The RET splice variant (c.1880-2A>C) found in both meningioma tumours is reported (rs193922699) as likely pathogenic in the Single Nucleotide Polymorphism Database (dbSNP). All missense variants detected in these two meningiomas are found in the cancer-driver genes. The eight variants we found in genes such as EGFR, PDGFRA, SMO, FLT3, PIK3CA, PTPN11, CDH1, and RB1 are glioma-driver genes. We did not find any mutations in genes such as BRAF, IDH1, CDKN2A, PTEN, and TP53, which are also listed as cancer-driver genes in gliomas. Mutation profiling utilising NGS technology in meningiomas could help in the accurate diagnosis and classification of these tumours and also in developing more effective treatments.

## Introduction

Meningioma tumours grow from the meninges surrounding the brain and spinal cord, which are usually benign. Meningiomas, or meningeal tumours, account for approximately 20%-30% of all intracranial tumours [1]. They are more common in women between the ages of 30 and 50 years, suggesting that female hormones may play a role in meningioma development [2-4]. Frequently, these tumours grow quite large before they can cause any symptoms, and the brain tries to adapt to their presence because of their slow growth [5-7]. According to their clinical and histopathological features, the World Health Organization (WHO) classified these tumours into three grades and 15 histopathological subtypes [8]. After complete resection, the five-year recurrence rates for grade 1 tumours are 5%-10% [9, 10].

Around 75%-90% of adult meningiomas are WHO-grade 1 tumours [11-13]. A vast range of morphologic features are reported in grade 1 meningiomas, and they are considered histologically benign, with fewer than four mitoses/10 microscopic high-power fields (HPF). According to the WHO categorization of benign meningiomas, there are nine types of meningiomas: meningothelial, fibroblastic, transitional (containing both meningothelial and fibroblastic components), psammomatous, angiomatous, microcystic, secretory, lymphoplasmacyte-rich, and metaplastic meningiomas. Psammomatous meningioma is the most common, involving the spine. These tumours occur more frequently in women between 50-70 years of age, and they present in the form of a calcified spinal lesion with half of the tumour mass constituting psammoma bodies [14]. Angiomatous meningioma is an uncommon histological variant of grade I meningiomas; only 2.1% of all meningiomas are this type. They demonstrate abundant blood vessels, usually greater than 50%, and the peritumoral oedema is widespread, affecting 75%-100% of the whole tumour. The main differential for an angiomatous meningioma is a hemangiopericytoma [15, 16].

Genetic conditions passed down through families, like neurofibromatosis type 2 (NF2) and multiple endocrine neoplasia type 1 (MEN1), can heighten the risk of developing meningiomas. Additionally, mutations that deactivate genes such as NF2 and meningioma 1 (MN1) are known to play a role in the advancement of meningiomas and their potential transformation into malignant forms [7,17,18]. Approximately 55% of meningiomas have inactivating mutations in the NF2 gene [19]. Additionally, in malignant meningiomas, whole exome sequencing (WES) identified several frequently mutated genes, including ARID1B, SEMA4D, and MUC2 mutations [17]. Next-generation sequencing (NGS) has recently changed the concept of the genetic status of meningiomas and demonstrated a clinical impact on meningioma diagnosis and molecular classification. The genotypes obtained by NGS analysis were significantly associated with clinicopathological features including histological subtypes, tumour size, MRI findings, and recurrence-free survival in meningioma cases [20, 21]. In 80% of the non-NF2 meningiomas, mutations in the TRAF7, v-AKT, AKT1, KLF4, SMO, and PIK3CA genes were identified by NGS analysis [19, 22, 23]. Most of the time, the loss of the NF2 gene and the mutations in the NF2 gene were mutually exclusive. Interestingly, none of the cases analysed in paediatric meningiomas showed an SMO or AKT mutation [24]. In angiomatous meningiomas, typical chromosomal polysomies of chromosomes 5, 13, and 20 were reported that were not present in other grade 1 meningiomas [25].

Numerous previous studies have focused on NGS in low-grade meningiomas; however, few studies have focused on mutation profiling in spinal meningiomas because of its rarity using NGS methodology. Thus, this study aimed to determine the mutation profile of a psammomatous meningioma, which is commonly found in spinal lesions, and an angiomatous (brain) meningioma tumour by NGS methodology on the Ion Proton System (Thermo Fisher Scientific, Waltham, MA). Our results provide a comparison of the mutational signatures of these tumours.

## Case presentation

Ethical approval

This study was approved by the bioethics committee of King Abdullah Medical City (KAMC), Makkah, Kingdom of Saudi Arabia (approval number: 14-140) and performed following the principles of the Declaration of Helsinki. Informed consent was obtained from the patient or the patient’s guardian before starting the study.

Tumours and tissue for analysis

The tumour was classified according to the WHO grading system [26]. The final diagnosis was made following radiological, histopathological, and immunological examinations. The first case of cranial meningioma (angiomatous) had a gross total resection (GTR) of the tumour, classified as Simpson grade 3, as the medial dura forming the lateral wall of the superior sagittal sinus was not coagulated. The second case of spinal meningioma (psammomatous), which was categorized as Simpson grade 2, had GTR of the tumour with coagulation of the adjacent dura. The formalin-fixed paraffin-embedded (FFPE) tumour tissue used in this NGS analysis was obtained from the Al-Noor Specialty Hospital, Makkah.

Radiology and histopathological analysis

A multi-slice CT (MSCT) using a 64-detector-row scanner performed the brain CT scan. Images were acquired with a 5mm slice thickness throughout on a GE LightSpeed volume CT (VCT) (GE Healthcare, Waukesha, WI) and 64-slice multidetector CT (MDCT). High-quality images were processed on the Volara^TM^ Digital Data Acquisition System (DAS) (GE Healthcare) at low-dose performance. A multi-sequential, multi-planar, and post-gadolinium MRI examination of the brain was performed on a Siemens 3T MAGNETOM Skyra MRI scanner (Siemens, Munich, Germany). 

Histopathological and immunological methods were performed as described in studies by Taher et al., Butt et al., and Jastania et al. [27-29]. Briefly, the FFPE sections were routinely stained using hematoxylin and eosin (H&E) on a Dako Coverstainer (Agilent Technologies, Inc., Santa Clara, CA) and immunohistochemistry using the Ventana BenchMark XT automated stainer (Roche Tissue Diagnostics, Basel, Switzerland), respectively. After staining, the images were processed using a Nikon Digital Microscope camera (DS-Ri1) (Nikon Instruments, Inc., Tokyo, Japan) with NIS Elements v.4.0 image software (Nikon Instruments, Inc.).

Isolation of DNA and NGS analysis on Ion Proton System

Details of DNA isolation and NGS analysis using the FFPE sections have been described in studies by Taher et al., Butt et al., Jastania et al., and Taher et al. [27-30]. Briefly, around 10 ng of DNA was used for NGS analysis, and the DNA was sequenced using an Ion PI v3 chip (Thermo Fisher Scientific) on an Ion Proton instrument sequencer Thermo Fisher Scientific). Libraries were prepared using the Ion AmpliSeq 2.0 library kit method (Thermo Fisher Scientific) and the Ion AmpliSeq cancer panel v.1 (Thermo Fisher Scientific), primer pools tagging with Ion Express barcodes (Thermo Fisher Scientific) [31]. More details of template preparation on OT2, enrichment methods, and sequencing on Ion Proton have been described previously [27-31]. After the sequencing, amplicon sequences were aligned to the human reference genome GRCh37 (hg19) in the target region of cancer panel v.1 gens using the Torrent Suite software v5.10.1.0 (Thermo Fisher Scientific). Variant call format (vcf) file was generated by running the Torrent Variant Caller Plugin v5.2. (Thermo Fisher Scientific). Filtering low-quality reads and the variant annotations were done on Ion Reporter software v5.10.2.0 (Thermo Fisher Scientific) using the vcf files. Details of data analysis and annotation sources are included in Ion Reporter software (Ion Reporter Software 5.6 Publication Number MAN0017204). The true mutations were considered only when the Phred score (i.e., -log10 of the probability that the alternative call is wrong) was above 20, and the significant mutation p-value was below 0.05.

Clinical presentation, radiology and histopathology

Case One

The first case is that of a grade 1 angiomatous meningioma patient. A male patient aged 62 years presented to the ER after an attack of a right upper limb partial seizure. He had been on aspirin since he had a cardiac catheterization two years ago. He had a history of right-sided weakness and a history of tongue-biting. The patient was conscious; on clinical examination, his Glasgow coma scale (GCS) was 15/15, and power was 5/5 bilaterally in all limbs, with no deformity. The patient was admitted, and the epileptic medication was started.

**Figure 1:**
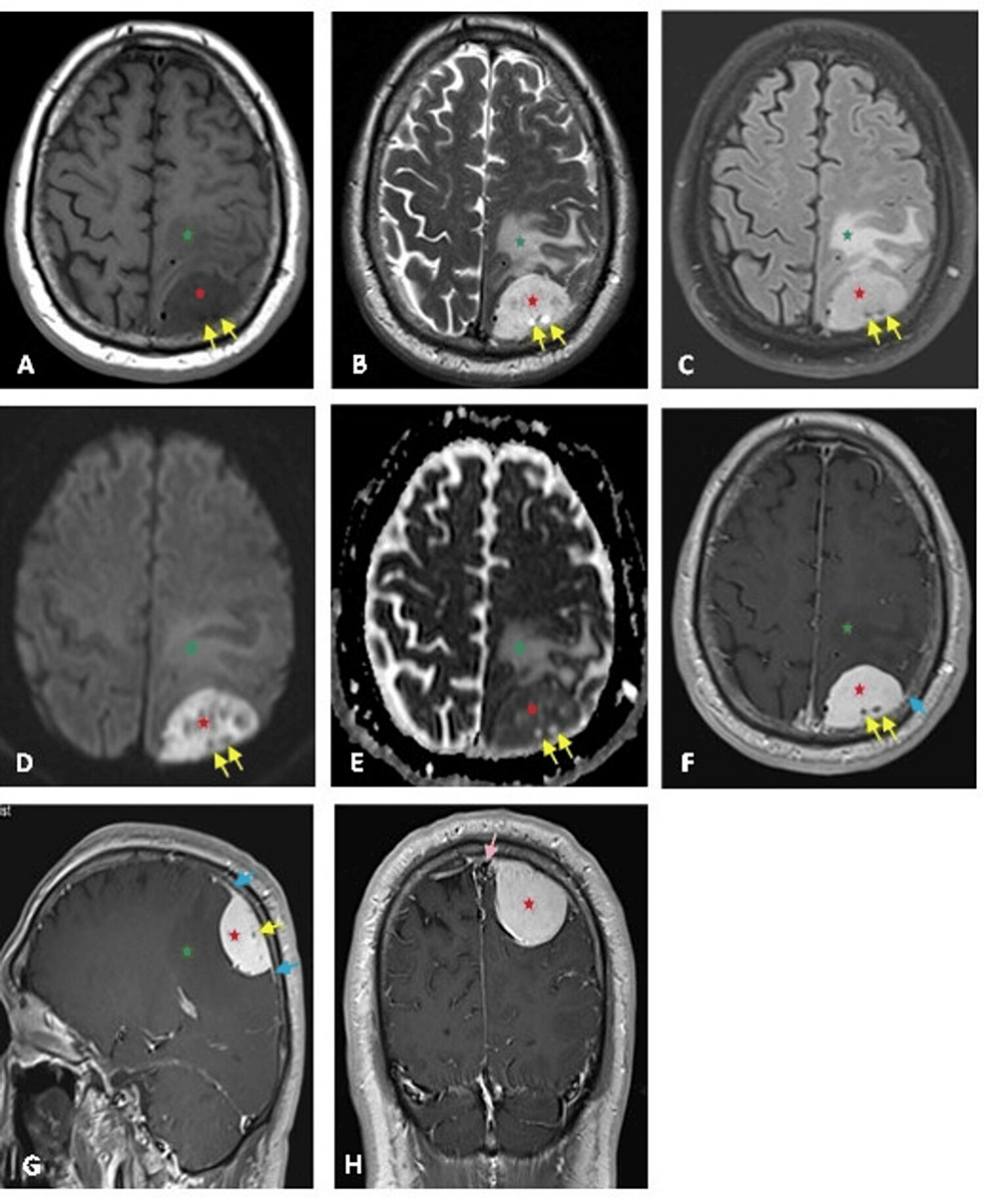
Brain MRI images of a 62-year-old male angiomatous meningioma patient MRI axial images T1WI (A), T2WI (B), T2TIRM (C), DWI (D), ADC map (E), and postcontrast axial T1 (F), sagittal T1 (G), and coronal T1 (H), showing a left parietal parasagittal, extra-axial lesion (red stars) with T1 hypointensity, heterogenous T2/T2TIRM hyperintensity, restricted diffusion on the DWI/ADC map, vivid postcontrast enhancement, dural tail (blue arrows), and non-enhancing cystic changes (yellow arrows). It is associated with vasogenic oedema (green star) compressing the adjacent cortex and adhering to the superior sagittal sinus (pink arrow). T1WI: T1 weighted image; T2WI: T2 weighted image; T2TIRM: T2 turbo inversion recovery magnitude; DWI: diffusion-weighted imaging; ADC: apparent diffusion coefficient of water

Magnetic resonance imaging (MRI) of the brain showed a left parietal parasagittal, extra-axial lesion with T1 hypointensity, heterogenous T2/T2 turbo inversion recovery magnitude (T2TIRM) hyperintensity, restricted diffusion on the diffusion-weighted imaging (DWI)/apparent diffusion coefficient of water (ADC) map, vivid postcontrast enhancement with dural tail, and non-enhancing cystic changes (Figure 1), suggestive of a meningioma.

It was associated with vasogenic oedema compressing the adjacent cortex and was adherent to the superior sagittal sinus without invasion or thrombosis.

The patient was operated on in a prone position under general anaesthesia (GA) and endotracheal intubation (ETT) with the head fixed in a Mayfield skull clamp. The left U-shaped parietal flap with its medial end at the midline was reflected laterally, and a free left parasagittal craniotomy was done. Microscopically, a dural flap was reflected medially over the superior sagittal sinus (SSS). The brain was oedematous even after the use of mannitol. The tumour was reddish, moderately vascular, soft, and well-differentiated from the brain by an arachnoidal plane. The tumour was dissected from the brain at the arachnoidal plane and removed in one piece. The lateral part of the dura over the tumour was removed, leaving the medial strip adjacent to the dural sinus, and then the hemostasis was done. The brain became more lax at the end of the surgery. A periosteal flap was used for the closure of the dural defect. The skull flap was fixed, and the scalp was closed in layers. The patient tolerated the surgery well and was discharged fully conscious and oriented without apparent neurological deficit.

**Figure 2:**
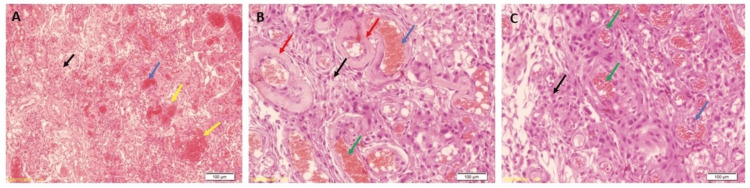
Hematoxylin and eosin-stained pictures of the 62-year-old male patient with angiomatous meningioma The tumour shows haemorrhage (blue arrow) and numerous vessels of variable sizes, with red blood cells (RBCs) inside the vessels (yellow arrow) and tumour cells (black arrow), (B) rich thin-walled (green arrow), along with hyalinized walls (red arrow), and (C) scattered meningothelial cells with pale eosinophilic cytoplasm and nuclei that are round to oval and show pseudo inclusion bodies (black arrow).

The tumour was confirmed to be angiomatous by the histopathological examination data (Figure 2).

**Figure 3:**
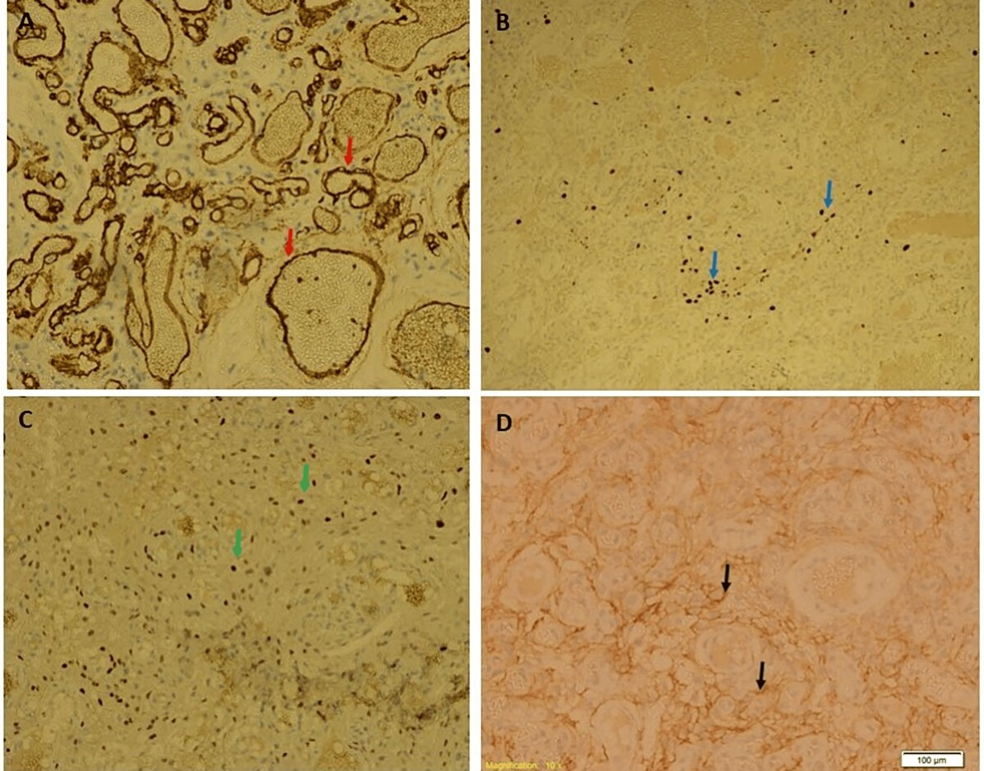
Immunohistochemistry pictures of an angiomatous meningioma tumour Immunostaining showing (A) CD-34 positive in blood vessels (red arrow), (B) Ki-67 showing low proliferation index (light blue arrow), (C) nuclear staining of progesterone receptor (PR) (green arrow), and (D) epithelial membrane antigen (EMA) positive in the membranes (black arrow)

Microscopic examination of the H&E-stained sections revealed neoplastic tissue composed of lobules of spindly-looking cells with pink cytoplasm running in short fascicles, forming syncytial structures and whorls. The cells were monotonous, had round to ovoid nuclei with fine chromatin and ample cytoplasm with indistinct borders, and showed clearing and pseudo-inclusions; >50% of the lesional area was composed of blood vessels. The vascular channels were small to medium-sized, had variable wall thickness, and were variably hyalinized. Moderate degenerative nuclear atypia was seen. Immunohistochemistry pictures are shown in Figure 3.

The tumour cells had a membranous expression of epithelial membrane antigen (EMA) and nuclear expression of progesterone receptor (PR). The tumour cells did not express CD34 (positive in blood cells only). The Ki-67 proliferative index was low.

**Figure 4:**
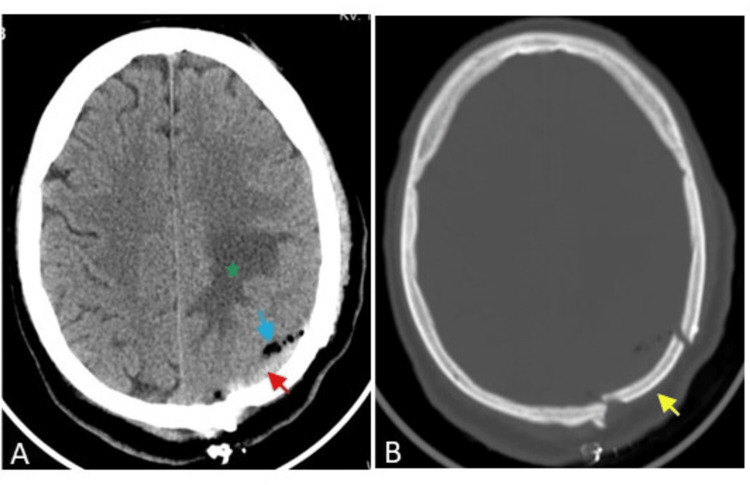
Postoperative brain CT scans (axial images) of the angiomatous meningioma patient Axial images of the brain (A) and bone (B) windows show a left parietal craniotomy (yellow arrow) with mild subdural acute hematoma (red arrow), pneumocephalus (blue arrow), and unchanged vasogenic oedema (green star).

A postoperative CT scan of the brain showed postoperative changes in the form of mild subdural acute hematoma, pneumocephalus with unchanged vasogenic oedema, and no evidence of the remnant tumour (Figure 4).

**Figure 5:**
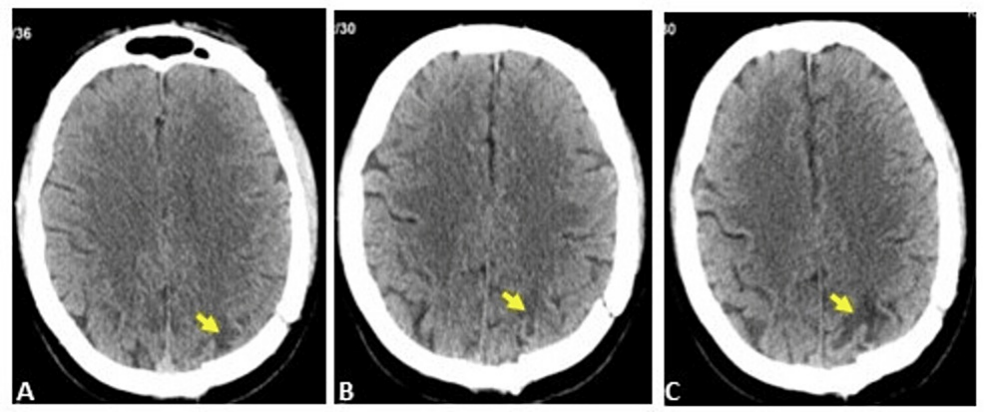
A serial follow-up CT scan of the brain of the angiomatous meningioma patient Axial images of the CT scan show new interval focal subcortical oedema (A) increasing in size (B-C).

He developed a pulmonary embolism on the third postoperative day, and a chest physician treated him. Later, he was discharged neurologically intact and advised to continue antiepileptic and anticoagulant medications and to follow up in neurosurgery and chest OPD. Four months after surgery, a follow-up brain CT scan showed a new interval of focal left parietal subcortical oedema adjacent to the surgical bed, increasing in size and hypodensity in the serial scans five and 10 months after surgery (Figure 5).

**Figure 6:**
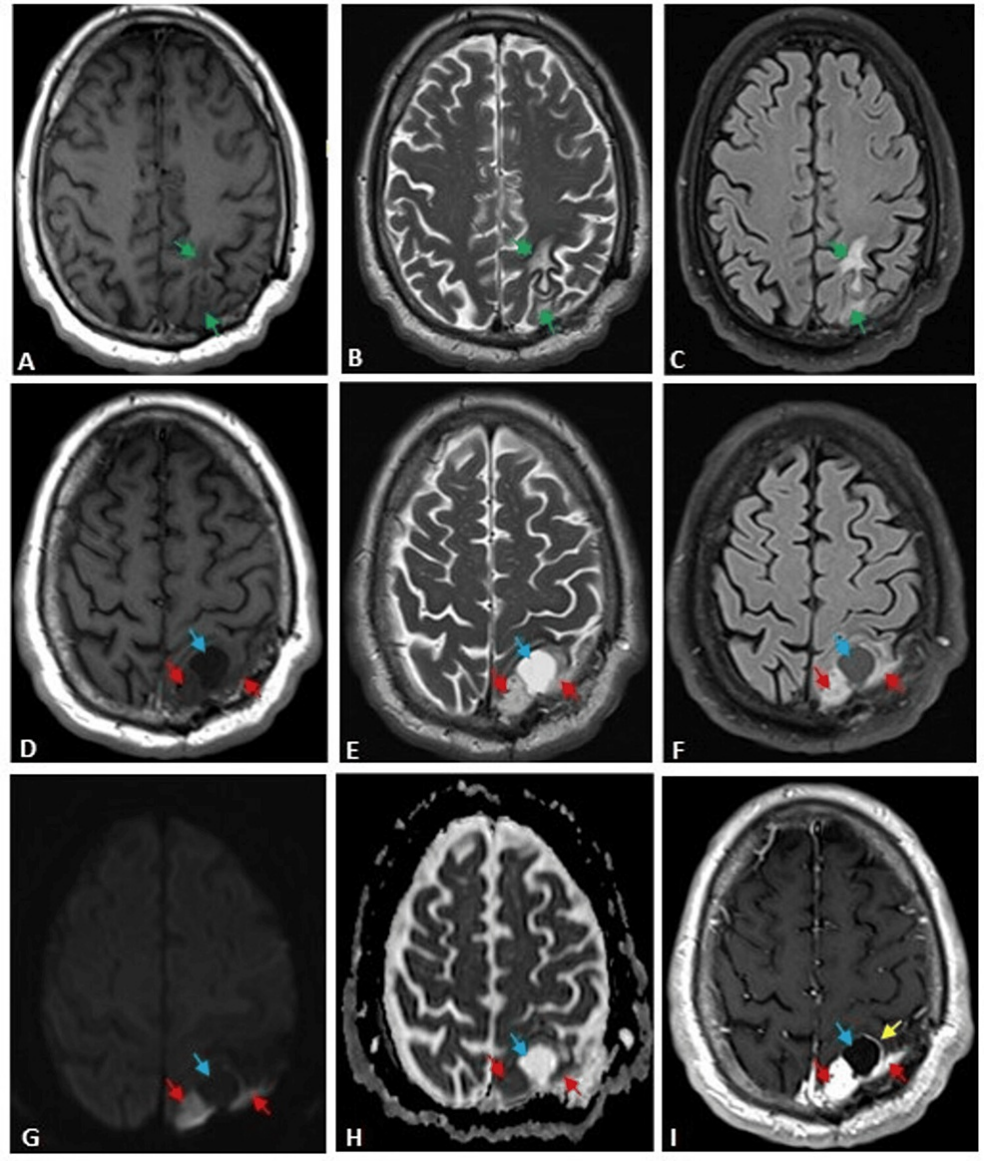
Follow-up brain MRI images of the angiomatous meningioma patient MRI axial images: T1WI (A, D), T2WI (B, E), T2TIRM (C, F), DWI (g), ADC map (H), and postcontrast T1 (I). The first row shows the left parietal subcortical white matter oedema. The second and third rows, just at the caudal level, show a left parietal parasagittal, extra-axial lesion at the site of the previous tumour with solid and cystic components. The solid component (red arrows) has T1 hypointensity, T2/T2TIRM hyperintensity, restricted DWI/ADC map diffusion, vivid postcontrast enhancement, and small signal void vessels. The cystic component (blue arrow) has T1/T2 isointensity relative to CSF, mild hyperintensity on T2TIRM, and an enhancing rim (yellow arrow). T1WI: T1 weighted image; T2WI: T2 weighted image; T2TIRM: T2 turbo inversion recovery magnitude; DWI: diffusion-weighted imaging; ADC: apparent diffusion coefficient of water; CSF: cerebrospinal fluid

The patient did not show up for regular follow-ups. After two years, an MRI of the brain with contrast showed a left parietal parasagittal, extra-axial lesion at the site of the previous tumour with solid and cystic components. The solid component had T1 hypointensity, T2/T2TIRM hyperintensity, restricted DWI/ADC map diffusion, vivid postcontrast enhancement, and small signal void vessels. The cystic component had T1/T2 iso intensity relative to CSF, mild hyperintensity on T2TIRM, and an enhancing rim, suggesting meningioma (Figure 6).

The plan was followed up for the recurrent tumour because of the medial location and relation to the major cortical venous drainage and superior sagittal sinus. However, the patient has not shown up since March 2021.

Case Two

**Figure 7:**
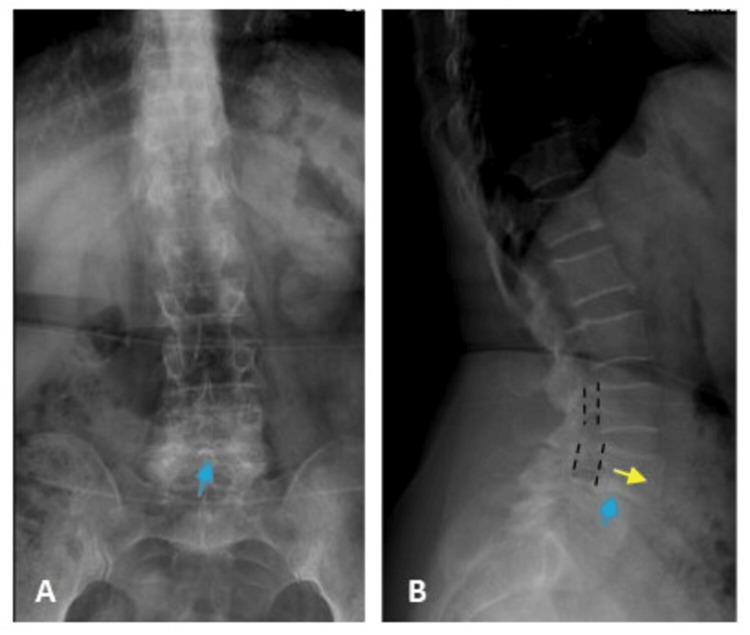
Lumbosacral radiographs of the psammomatous meningioma patient X-ray images of anteroposterior (A) and lateral (B) views show grade 2 spondylolisthesis of L4 over L5 (yellow arrow) with the widening of the spinal canal (black lines) and severe narrowing of L4/L5 intervertebral disc space (blue arrow).

The second case was of a 64-year-old female who presented to the ER with progressive weakness in both lower limbs for three years. When she was diagnosed with a spinal tumour, she was advised to have surgery, but she refused. She had become unable to walk, so she sought medical advice again. Clinically, she was fully conscious; the motor examination revealed that the patient had normal power in both upper limbs (3/5 and 2/5 for the right and left lower limbs, respectively). The sensory level was at L1. Initial lumbosacral radiographs showed grade 1 anterior displacement of L4 over L5 with the widening of the spinal canal and severe narrowing of intervertebral disc space (Figure 7).

**Figure 8:**
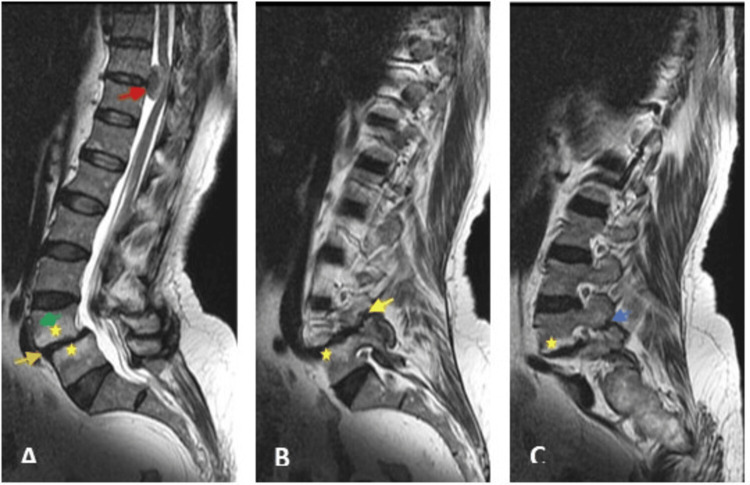
Sagittal MRI images showing spondylolisthesis in the psammomatous meningioma patient Sagittal MRI images T2WI (A), T1WI (B), and (C) showing grade 2 spondylolisthesis of L4 over L5 vertebral bodies (green arrow) with right (yellow arrow) and left (blue arrow) pars interarticularis defects, associated with severe disc degeneration (orange arrow) at L4/L5 level, and adjacent vertebral endplate changes Modic type 2 (yellow stars). An intradural mass (red arrow) compressing the spinal cord is incidentally seen at the D10/D11 level. T2WI: T2 weighted image; T1WI: T1 weighted image

An MRI of the lumbosacral spine (Figure 8) showed bilateral pars interarticular defects causing grade 1 spondylolisthesis of L4 over L5, associated with severe degenerative disc changes and adjacent Modic type 2 vertebral endplate changes.

**Figure 9:**
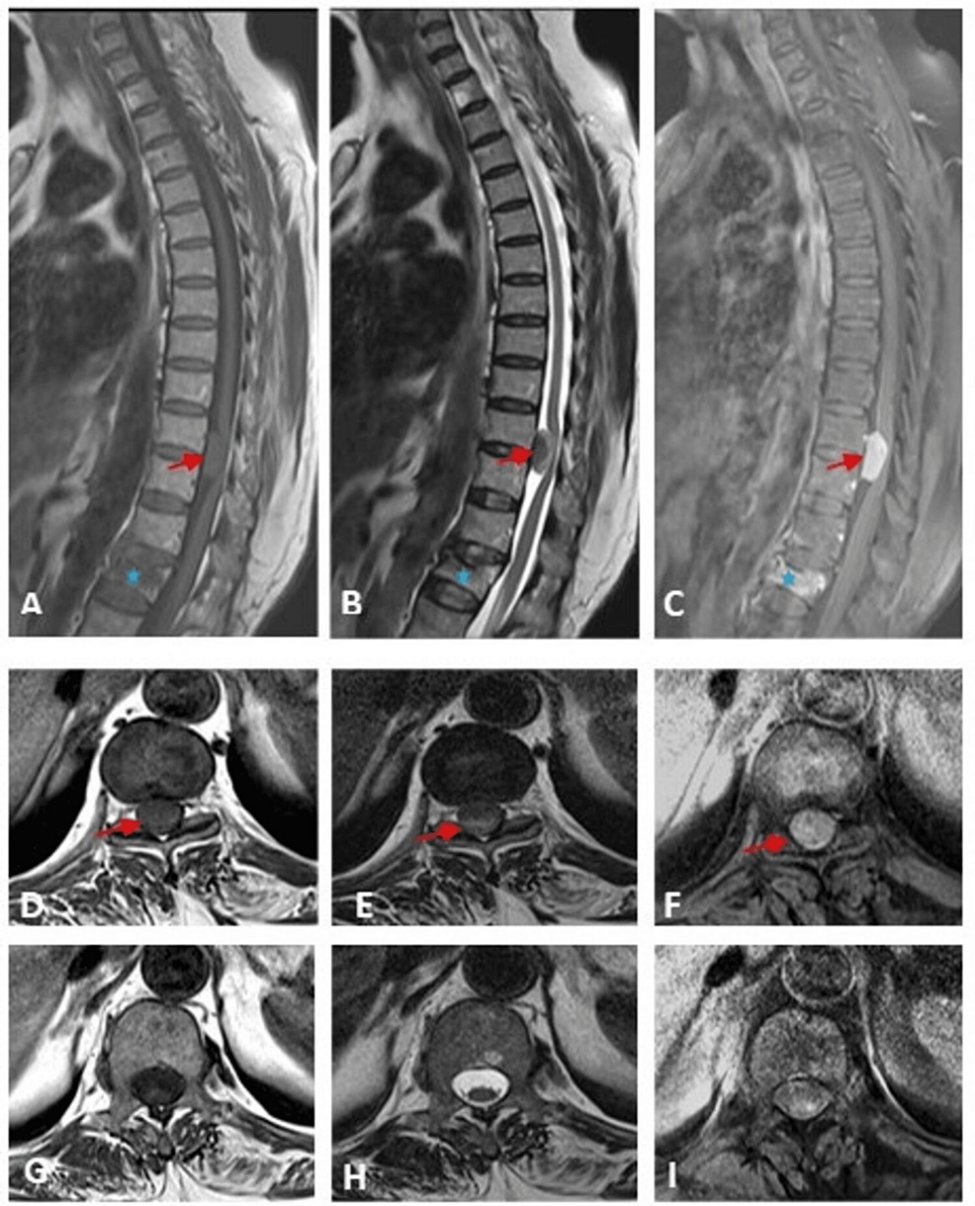
The MRI of the thoracic spine of a patient with psammomatous meningioma revealed an intradural extramedullary lesion. MRI scan images T1WI (A, D, G), T2WI (B, E, H), postcontrast T1 (C, F, I), sagittal images (A to C), and axial images at the level of the lesion (D, E, F), and caudal images (G, H, I), showing an intradural extramedullary lesion at the D10/D11 level (red arrows) compressing the spinal cord. The lesion shows isointensity relative to the spinal cord on T1WI, hypo- and hyperintensity on T2WI, and vivid postcontrast enhancement. A new interval acute compression fracture of the L1 vertebral body (blue star). T1WI: T1 weighted image; T2WI: T2 weighted image

An intradural extramedullary mass was seen at the D10/D11 level, compressing the spinal cord and needing further evaluation by MRI dorsal spine with contrast. The patient was advised to undergo surgery to excise the tumour, but the patient refused and showed up after three years as she was unable to walk. An MRI of the thoracic spine (Figure 9) showed an intradural extramedullary lesion at the D10/D11 level of T1 isointensity relative to the spinal cord, T2 hypo- and hyperintensity, and vivid postcontrast enhancement, suggestive of a meningioma.

A new interval acute compression fracture of the L1 vertebral body was seen.

The patient was operated on in a prone position under GA and ETT. The T10-T11 level was confirmed using a C-arm X-ray. A midline skin incision was made, then paraspinal muscles were stripped off the spinous processes and laminae of T10 and T11 vertebrae, and then the level was reconfirmed with a C-arm X-ray.

The T10 and T11 laminectomies were done. Then, microscopically, the dura was opened and hitched; the spinal cord appeared to be bulging. Through the left posterolateral gutter, the tumour was seen and centrally debulked, then dissected off the cord via the arachnoid plane, and then completely removed in a piecemeal fashion. The tumour was moderately vascular, with areas of calcifications. The dura was closed after good hemostasis, and the wound was closed in layers.

**Figure 10:**
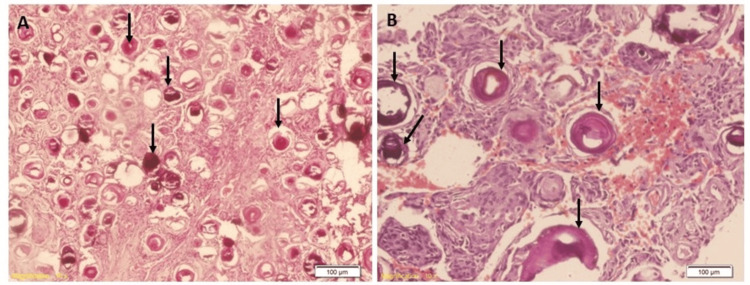
Hematoxylin and eosin-stained pictures of the psammomatous meningioma tumour Histological examination of the resected tumour is characterised by the abundance of calcified psammoma bodies (black arrows) and inconspicuous meningothelial or fibroblastic-like cells.

The patient tolerated the surgery well. In the following days, she gradually recovered motor and sensory functions in both lower limbs before discharge. Microscopic examination of H&E sections revealed meningothelial proliferation with patternless, sheet-like architecture and inconspicuous whorls and lobules. The cells were round to ovoid, harbouring fine chromatin and inconspicuous nucleoli. Intranuclear pseudo-inclusions were also noted. Numerous psammoma bodies were seen (Figure 10).

**Figure 11:**
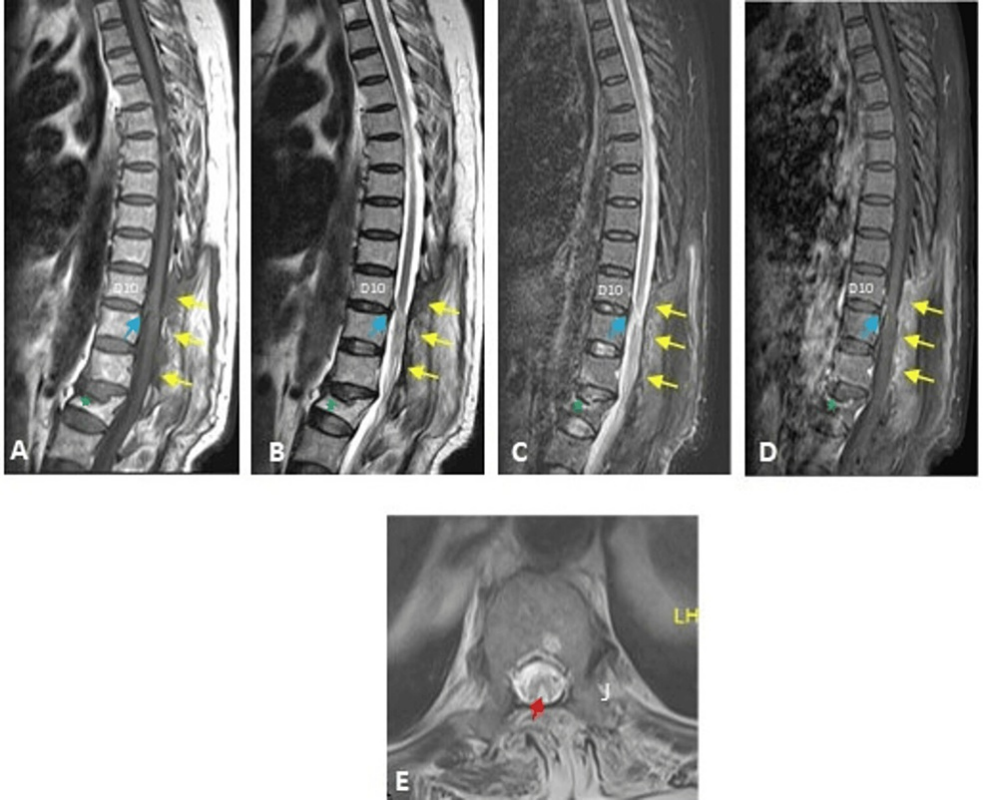
Follow-up MRI images showed postoperative laminectomies with complete resection of the previous intradural extramedullary lesion in the psammomatous meningioma patient MRI T1WI (A), T2WI (B, E), STIR (C), postcontrast T1 (D), sagittal images (A to D), and axial (E) images, showing postoperative laminectomies (yellow arrows) at D10 and D11 levels with complete resection of the previous intradural extramedullary lesion at D10/D11 level, focal enhancing dural thickening (blue arrows), and myelomalacia (red arrow). Compression fracture (anterior wedging) of the L1 vertebral body (green stars). T1WI: T1 weighted image; T2WI: T2 weighted image; STIR: short tau inversion recovery

Psammoma bodies are widespread in spinal meningiomas [32]. A follow-up MRI showed postoperative laminectomies at D10 and D11 levels with complete resection of the previous intradural extramedullary lesion at D10/D11 levels and myelomalacia in addition to anterior wedging of the L1 vertebral body (Figure 11).

Next-generation sequencing data analysis for variant identification

The quality assessment metrics of the chip run on an Ion Proton instrument generated by Torrent Suite software were reported by Taher et al. recently [31]. In that, bead loading has been shown. The Ion Sphere™ Particle (ISP) density image shows percentage loading across the physical surface, the final library, adapter dimers, the percentage of chip wells that contain live ISPs, total reads, usable reads, aligned and unaligned reads, total bases, key signal, and ISP summary details such as loading percentage, enrichment (polyclonal and clonal), etc. [31].

Alignment to the reference genome (hg 19) with the target regions (CP.20131001.designed) and coverage overview for the two tumours as performed by the Ion Torrent Suite software v4.4.2 are shown in the appendix (Appendix A). A total of 6,211,098 and 7,097,200 reads were generated for psammomatous and angiomatous tumours using the Ion PI v.3 chip, with 84.31% and 84.30% reads being on target for these tumours, respectively. Base read coverages of sequencing are shown for amplicons and targets in the appendix (Appendices B-C). One hundred and eighty-nine amplicons were sequenced out of the 190 total amplicons present in this custom panel primer pool. The 1x, 20x, and 100x target base coverage was 100%, and the 500x target base coverage was 98.32% and 96.33% for psammomatous and angiomatous tumours, respectively. End-to-end reads of amplicons were above 96% for both tumours (Appendix C).

Common exonic variants detected by the Ion Reporter software between psammomatous and angiomatous meningiomas are shown in Table 1.

**Table 1:** Exonic (missense) variants common between psammomatous and angiomatous meningiomas AA: amino acid; cDNA: coding DNA

Chromosomal position	Ref	Psammomatous-observed	Angiomatous-observed	Frequency	Gene	AA change	cDNA changed	Novel/reported
chr2:29432738	A	C/C	C/C	1	ALK	p. (Ile1250Met)	c.3750T>G	rs760315884
chr3:10191554	T	G/G	G/G	1	VHL	p. (Ser183Ala)	c.547T>G	Novel
chr3:41266152	G	T/T	T/T	1	CTNNB1	p. (Gly50Val)	c.149G>T	Novel
chr7:55233069	G	GC/GC	GC/GC	1	EGFR	p. (Val607fs)	c.1819_1820insC	rs397517105
chr7:128845046	T	T/G	G/G	1	SMO	p. (Asn180Lys)	c.540T>G	Novel
chr7:128846102	A	A/T	A/T	1	SMO	p. (Lys344Asn)	c.1032A>T	Novel
chr9:133747566	A	C/C	C/C	1	ABL1	p. (Lys291Asn)	c.873A>C	Novel
chr11:533805	AT	AT/A	AT/A	1	HRAS	p. (Ile84fs)	c.250delA	Novel
chr11:108204613	A	A/G	A/G	1	ATM	p. (Lys2643Arg)	c.7928A>G	Novel
chr12:121431997	AT	AT/A	AT/A	1	HNF1A	p. (Ser249fs)	c.745delT	Novel
chr13:28610096	T	T/A	T/A	1	FLT3	p. (Lys465Met)	c.1394A>T	Novel
chr13:49039243	C	C/A	A/A	1	RB1	p. (Thr774Asn)	c.2321C>A	Novel

Ten novel variants and two reported variants were common between these two tumours. Nine variants were missense, an insertion in EGFR c.1819_1820 insCA, causing frameshifting; and a single nucleotide deletion in HRAS and HNF1A genes, respectively, which caused frameshifting in these genes (Table 1).

Common intronic and synonymous variants detected between psammomatous and angiomatous meningiomas are shown in Table 2.

**Table 2:** Common intronic and synonymous variants detected between psammomatous and angiomatous meningiomas AA: amino acid; cDNA: coding DNA

Chromosomal position	Ref	Psammomatous-observed	Angiomatous-observed	Frequency	Location	AA changed	cDNA changed	Novel/reported
chr2:29432735	A	T/T	T/T	1	ALK: exonic	p. (Ala1251Ala)	c.3753T>A	Novel
chr2:209113084	A	A/T	A/T	1	IDH1: intronic	p.?	c.414+9T>A	Novel
chr3:37067193	G	T/T	G/T	1	MLH1: exonic	p. (Ser368Ser)	c.1104G>T	rs769364808
chr4:1807894	G	A/A	A/A	1	FGFR3: exonic	p. (Thr653Thr)	c.1953G>A	rs7688609
chr4:55141055	A	G/G	G/G	1	PDGFRA: exonic	p. (Pro567Pro)	c.1701A>G	rs1873778
chr4:55144199	A	A/C	A/C	1	PDGFRA: intronic	p.?	c.2002+26A>C	Novel
chr4:55599265	T	C/C	C/C	1	KIT: exonic	p. (Asn797Asn)	c.2391T>C	Novel
chr4:55972896	T	C/C	C/C	1	KDR: exonic	p. (Glu498Glu)	c.1494A>G	Novel
chr4:55972997	G	C/C	C/C	1	KDR: intronic	p.?	c.1413-20C>G	Novel
chr5:112175770	G	A/A	G/A	1	APC: exonic	p. (Thr1493Thr)	c.4479G>A	rs41115
chr7:55248981	T	C/C	T/C	1	EGFR: intronic, EGFR-AS1: exonic_nc	p.?	c.2284-5T>C	Novel
chr7:128846435	G	A/A	A/A	1	SMO: intronic	p.?	c.1264+7G>A	Novel
chr10:43609926	A	A/C	A/C	1	RET: splicesite_5:	p.?	c.1880-2A>C	rs193922699
chr10:43613843	G	G/T	G/T	1	RET: exonic	p. (Leu769Leu)	c.2307G>T	rs1800861
chr10:89711952	A	A/C	A/C	1	PTEN: exonic	p. (Pro190Pro)	c.570A>C	Novel
chr11:108170535	T	A/A	A/A	1	ATM: exonic	p. (Leu1700Leu)	c.5100T>A	Novel
chr11:108225536	AG	AG/GT	GT/GT	1	ATM: splicesite_5:	p.?	c.8787-2AG>GT	Novel
chr12:121431300	C	C/T	C/T	1	HNF1A: intronic	p.?	c.527-23C>T	rs1169301
chr14:105246389	CAAA	ATCA/CATG	ATCA/CATG	1	AKT1: intronic	p.? p.?	c.175+36TTG>GAT, c.175+34TT>CA	rs750000813
chr17:37881506	A	A/T	A/T	1	ERBB2: intronic	p.?	c.2649+49A>T	Novel

Ten synonymous variants and 10 intronic variants were common between these two tumours. Twelve were novel variants, and eight were reported variants common between these two tumours. Two splice site_5’ variants (acceptor site variants) were common in these two tumours. In ATM, a splice site_acceptor (c.8787-2AG>GT) was novel, and in RET (c.1880-2A>C) was reported previously (rs193922699).

Tables 3-4 present the exonic variants in angiomatous and psammomatous meningiomas typical of these tumours, respectively.

**Table 3:** Exonic variants typical of angiomatous meningioma cDNA: coding DNA; AA: amino acid

Chromosomal position	Ref	Observed allele	Gene	cDNA changed	Frequency (%)	AA change	Novel/reported	Exon
chr2:212530182	C	A	ERBB4	c.1737G>T	80	p. (Lys579Asn)	rs377501321	15
chr3:178921473	A	T	PIK3CA	c.955A>T	66.67	p. (Asn319Tyr)	Novel	5
chr3:178938877	G	A	PIK3CA	c.2119G>A	20.75	p. (Glu707Lys)	rs3729687	14
chr3:178952020	C	T	PIK3CA	c.3075C>T	49	p. (Thr1025Thr)	rs17849079	21
chr4:55144609	A	G,T	PDGFRA	c.2083A>G, c.2083A>T	G=72.00, T=20.00	p. (Ser695Gly), p. (Ser695Cys)	Novel	15
chr4:55594198	G	A	KIT	c.1901G>A	100	p. (Arg634Gln)	rs766264502	13
chr4:55595578	A	T	KIT	c.2068A>T	80	p. (Ile690Phe)	Novel	14
chr4:55602766	T	C	KIT	c.2587T>C	60	p. (Phe863Leu)	Novel	18
chr4:55602768	C	G	KIT	c.2589C>G	50	p. (Phe863Leu)	Novel	18
chr4:55979579	TC	T	KDR	c.867delG	100	p. (Ser290fs)	Novel	7
chr4:55979585	A	G	KDR	c.862T>C	94.74	p. (Phe288Leu)	Novel	7
chr9:133750312	T	C	ABL1	c.1143T>C	66.67	p. (Asp381Asp)	Novel	7
chr11:108172493	C	G	ATM	c.5296C>G	100	p. (Pro1766Ala)	rs778309703	35
chr12:112888128	T	A	PTPN11	c.144T>A	76.92	p. (Asn48Lys)	Novel	3
chr16:68835705	TG	T, TT	CDH1	c.297G>T, c.298delG	T=28.57, TT=57.14	p. (Leu99Phe), p. (Val100fs)	Novel	3
chr18:48604771	G	T	SMAD4	c.1593G>T	95.59	p. (Arg531Arg)	Novel	12
chr22:24145501	GC	G	SMARCB1	c.521delC	98.31	p. (Ala174fs)	Novel	5

**Table 4:** Exonic variants typical of psammomatous meningioma cDNA: coding DNA; AA: amino acid

Chromosomal position	Ref	Observed allele	% Frequency	Gene	cDNA changed	AA changed	Novel/reported	Exon
chr2:212288904	C	T	46.88	ERBB4	c.2842G>A	p. (Asp948Asn)	Novel	23
chr4:55144092	C	G	41.67	PDGFRA	c.1921C>G	p. (Leu641Val)	Novel	14
chr4:55972946	A	G	45.61	KDR	c.1444T>C	p. (Cys482Arg)	rs34231037	11
chr4:55972974	T	A	48.25	KDR	c.1416A>T	p. (Gln472His)	rs1870377	11
chr5:112175210	ATA	A, AA, AAA	A=0.78, AA=99.22, AAA=0.00	APC	c.3920delT	p. (Ile1307fs)	rs121913224, rs1801155, rs763847228	16
chr5:112175277	AC	AT, GT	AT=50.00, GT=37.50	APC	c.3986_3987delACinsGT, c.3987C>T	p. (His1329Arg), p. (His1329His)	Novel	16
chr9:133738399	A	C	80	ABL1	c.799A>C	p. (Thr267Pro)	Novel	4
chr10:123279560	A	C	84.62	FGFR2	c.872T>G	p. (Ile291Ser)	Novel	7
chr12:121431340	C	T	34.74	HNF1A	c.544C>T	p. (Gln182Ter)	Novel	3
chr12:121431365	C	T	32.99	HNF1A	c.569C>T	p. (Thr190Ile)	Novel	3
chr12:121431373	G	A	18.5	HNF1A	c.577G>A	p. (Glu193Lys)	rs766410121	3
chr19:1207121	A	G	100	STK11	c.209A>G	p. (Glu70Gly)	Novel	1
chr19:17947972	G	C	100	JAK3	c.1752C>G	p. (Leu584Leu)	rs200582253	13

In the angiomatous type, 11 novel and six previously reported variants were found; three were synonymous, 11 were missense mutations, and three were deletions causing frameshifting (Table 3). The deletion variants were in the SMARCB1, CDH1, and KDR genes. The SMARCB1, KDR, and CDH1 deletion variants were novel. These variants were not detected in psammomatous meningioma tumours.

In the psammomatous meningioma tumour, eight novel and five previously reported variants were found (Table 4). In this tumour, two variants were synonymous: a deletion causing a frameshifting in [(c.3920delT; p. (Ile1307fs)], and a two-base pair insertion and deletion (INDEL) [(c.3986_3987delACinsGT; p. (His1329Arg)] both in the APC gene were also found (Table 4). Unique intronic variants found in psammomatous and angiomatous meningioma tumours are shown in Tables 5-6.

**Table 5:** Intronic variants typical of angiomatous meningioma cDNA: coding DNA; AA: amino acid

Chromosomal position	Reference	Observed allele	Frequency (%)	Gene	cDNA changed	AA changed	Novel/reported	Exon
chr2:29432628	A	C	100	ALK	c.3836+24T>G	p.?	Novel	
chr7:128845982	T	A	71.43	SMO	c.921-9T>A	p.?	Novel	
chr7:128845984	T	A	71.43	SMO	c.921-7T>A	p.?	Novel	
chr9:22006264	G	A	96.49	CDKN2B, CDKN2B-AS1	c.157-18C>T	p.?	rs776463289	
chr10:89624217	G	T	50	PTEN	c.-10G>T	p.?	Novel	1
chr10:89717788	A	T	37.5	PTEN	c.801+12A>T	p.?	rs75603592	
chr12:25380365	A	G	95.56	KRAS	c.112-19T>C	p.?	Novel	
chr14:105246407	G	A	42.75	AKT1	c.175+18C>T	p.?	ClinVar Miner	
chr22:24145615	A	G	100	SMARCB1	c.628+6A>G	p.?	rs2287120	

**Table 6:** Intronic variants typical for psammomatous meningioma cDNA: coding DNA; AA: amino acid

Chromosomal position	Reference	Observed allele	Percentage Frequency	Gene	cDNA changed	AA changed	Novel/reported
chr4:55144201	TT	GC	100	PDGFRA	c.2002+28TT>GC	p.?	Novel
chr4:55561656	A	G	30.77	KIT	c.68-22A>G	p.?	rs746132062
chr4:55960966	T	C	66.67	KDR	c.2971+3A>G	p.?	Novel
chr5:149433596	TG	GA	100	CSF1R1	c.*1841TG>GA	p.?	rs2066933
chr9:133748224	A	T	50	ABL1	c.908-23A>T	p.?	Novel
chr13:28608185	TG	GG	GG=45.45	FLT3	c.1837+34A>C	p.?	Novel
chr16:68846170	A	G	66.67	CDH1	c.1137+4A>G	p.?	Novel
chr17:7578306	A	T	55.56	TP53	c.560-17T>A	p.?	Novel
chr18:48593385	T	A	40	SMAD4	c.1140-4T>A	p.?	Novel

Five novel and four previously reported variants were detected in angiomatous meningioma, which were not found in the psammomatous meningioma (Table 5). In contrast, seven novel and two previously reported variants were found in psammomatous tumours, which were not in angiomatous tumours (Table 6).

The PolyPhen-2 score (Harvard University) predicts the impact of substituting another amino acid on the protein structure and function. This score represents that a substitution is damaging if it is near 1.0, and a score closer to 0.0 tolerates the impact of the mutation. In contrast, a variant with a sorting intolerant from tolerant (SIFT) score of 0.0 predicts that this substitution is pathogenic. The PolyPhen-2, SIFT, and Phred scores and the p-values of exonic variants of psammomatous and angiomatous tumours are shown in Appendices D-E. In the angiomatous meningioma, the KIT variant p. (Arg634Gln) has a SIFT score of 0.01 and a PolyPhen2 score of 1, which indicates it’s pathogenic. The other two variants of KIT, p. (Phe863Leu) and p. (Phe863Leu), also had 0.0 and 0.97 scores of SIFT and PolyPhen2, indicating these are also pathogenic. The CDH1 p. (Val100fs), ERBB4 p. (Lys579Asn) variants, and two variants in PIK3CA [p. (Asn319Tyr), and p. (Glu707Lys)] have PolyPhen2 scores of 0.0915, 0.911, 0.965, and 0.998, respectively, as shown in Appendix D, which suggests these are also pathogenic. In the variant analysis on Ion Reporter software, a true mutation was considered based on a Phred quality score >20, and mutations with a p-value <0.05 were considered significant. Variants that do not fall under this criterion were filtered out.

## Discussion

Several investigations in the past have focused on identifying mutations in meningioma genes and trying to correlate them with their aggressiveness using next-generation DNA sequencing techniques. It was reported that 10% of sporadic meningiomas exhibit AKT1 mutations, causing constitutive AKT1 activation [33]. Yuzawa et al. have reported that out of 103 meningioma tumours, at least 81 tumours have at least one or more mutations in genes such as NF2, TRAF7, AKT1, KLF4, and SMO, and/or loss of NF2 [21,9]. We did not detect any AKT1 mutations in these meningiomas. However, in an angiomatous tumour, we found a novel p. (Asn319Tyr) and a known p. (Glu707Lys) missense mutation in the PIK3CA gene, which is not present in the psammomatous tumour. Previously, Jastania et al. reported in the adamantinomatous craniopharyngioma (ACP) tumour PIK3CA mutations [(p.Ile391Met) and p. (Met1043Thr)] but they are different than what we found in this angiomatous meningioma tumour [29]. Bi et al. have reported that PIK3CA mutations are prevalent in low-grade meningiomas, and NF2 mutations and several genomic alterations are distinctive of high-grade meningioma tumours [34,35]. Moreover, it was reported that, in 14 angiomatous meningiomas tested by NGS analysis by Abedalthagafi et al., no mutations were detected in cancer-driver genes such as AKT1, SMO, KLF4, and PIK3CA [25]. It was reported that SMO and AKT1 mutations are common, and the AKT1, SMO, and PIK3CA mutations are mutually exclusive [22, 23, 36]. Interestingly, a missense mutation p. (Ser183Ala) was found in both tumours of meningioma in the VHL gene in this investigation, which is frequently mutated in hemangioblastoma (HB) tumours [37]. FGFR3 mutations were reported in meningiomas for the first time by AlSahlawi et al. [38]. We did not detect FGFR3 mutations in these two meningiomas; however, an FGFR2 missense mutation was found in a psammomatous meningioma.

Previously, we reported the SMO variant p. (Asn180Lys) in the HB tumour. In the present investigation, this mutation and an additional variant p. (Lys344Asn) were detected in both meningiomas. HNF1A p. (Ser249fs) and ALK p. (Ile1250Met) variants found in both meningioma tumours were also previously found in HB tumours. CTNNB1 mutations are not commonly found in meningiomas. In the COSMIC database, a c.149G>A, [p. (Gly50Val) (COSM5718)] mutation was reported in the CTNNB1 gene, causing the same missense change p. (Gly50Val); however, we found a c.149G>T change, which is a novel one in these meningioma tumours. This mutation is present in exon 3 of the CTNNB1 gene, and mutations present in this exon are known to drive tumorigenesis in many cancers. For example, ACP tumours exhibit activating mutations in the casein kinase-1 (CK1-alpha) and glycogen synthase kinase-3 (GSKE-3) phosphorylating domains of the CTNNB1 gene in exon 3, between amino acids 32-45, targeting the degradation of the CTNNB1 protein complex [29]. Of note, we have previously identified the PIK3CA, c.2119G>A, p. (Glu707Lys); KDR c.862T>C, p. (Phe288Leu) variants found in angiomatous meningioma in HB tumour as well [31]. 

Synonymous variants found in the ALK, MLH1, FGFR, PDGFR, KIT, KDR, APC, RET, PTEN, and ATM genes are common in these two tumours. The CSF1R 3’-variant (rs2066933) found in psammomatous meningioma has been reported by Taher et al. in a myxopapillary ependymoma (MPE) as well[30]; PDGFRA synonymous variant p. (Pro567Pro) and FGFR3 p. (Thr653Thr) found in both meningiomas were previously detected in anaplastic ependymoma grade 3, ACP, and MPE tumours also [28,29,30]. The synonymous variants detected in both meningiomas, such as in FGFR3 p. (Thr653Thr), PDGFRA p. (Pro567Pro), and APC p. (Thr1493Thr), respectively, were also reported in atypical choroid plexus papilloma tumours [27].

We have detected a SMARCB1 variant [c.521delC; p. (Ala174fs)] in the angiomatous tumour. However, in this amino acid codon, another variant c.520G>T [p. (Ala174Ser), (rs746433763)] was reported in the ClinVar database, accession number RCV000794971.5. The tumour suppressor protein SMARCB1/IN11 is part of the SWI/SNF transcriptional complex. Expression of this protein is entirely absent in malignant rhabdoid tumours [39]. Previous studies have shown germline mutations in familial multiple meningiomas and somatic mutations in solitary meningiomas. Those mutations were in exon 2 and exon 9 of the SMARCB1 gene [19,40,41,42]. Clark et al. have reported a somatic mutation of SMARCB1 in one out of 50 meningiomas [22]. Other investigators have reported that only one of the 80 meningiomas showed an insertion mutation in this gene, and only four out of 126 tumours (3%) had mutations, suggesting that the INI1 mutation is a rare event in the molecular pathology of meningiomas, in contrast to the role it plays in the development of schwannoma [41-44].

In the ATM gene, a pathogenic splice variant c.7928-2A>G (variant #0000401978) was reported in variants in the Leiden Open Variation Databases (LOVD) (http://www.lovd.nl/3.0/home). This is at the exon 53 position; we have detected a missense variant in this codon [c.7928 (A>G), p. (Lys2643Arg)] in both tumours, which is a novel one. Another ATM, splice site_5 variant detected in both tumours is also a novel one (c.8787-2AG>GT); however, in LOVD, another mutation is also reported (variant #110376) at this codon (c.8787-1G>T). STK11 mutations were reported in 3.7% of both low- and high-grade meningiomas [19, 45]. Increased mortality was reported in patients whose meningioma harboured an STK11 mutation [45].

Several mutations in gastrointestinal meningiomas have been reported through NGS using the 50-gene AmpliSeq Hotspot Cancer Panel v2 (Thermo Fischer Scientific) [46]. The most frequently mutated genes were c-KIT, ATM, TP53, EGFR, STK11, NRAS, SMAD4, FGFR3, and PTPN11; less frequent mutations were SMARCB1, FLT3, KRAS, FBWX7, ABL1, ERBB2, IDH1, BRAF, MET, HRAS, RB1, CTNNB1, PIK3CA, VHL, KDR, APC, NOTCH1, JAK3, and SRC. All missense variants detected in these two meningiomas in our investigation are in the cancer-driver genes (https://www.intogen.org/search). And the eight variants, viz., EGFR, PDFGRA, SMO, FLT3, PIK3CA, PTPN11, CDH1, and RB1, are also in the glioma-driver genes (https://www.intogen.org/search?cancer=GBM). These mutations can enhance diagnostic accuracy and clinical decision-making.

## Conclusions

We reported several novel and previously known variants in two meningioma tumours identified by NGS technology. Also, we have identified common and typical mutations for psammomatous meningioma and angiomatous meningioma, respectively. All missense variants detected in these two meningiomas are found to be present in cancer-driver genes or glioma-driver genes in different databases. These mutations can enhance diagnostic accuracy and clinical decision-making and probably guide the development of treatment plans in the selected patients with meningiomas with hedgehog, WNT, and PIK3CA signalling inhibitors. Missense mutations in other cancer-driver genes, such as PTEN, TP53, IDH1, NPM1, and CDKN2A, which are frequently affected in gliomas, were not found in the tumours of this study.

## Figures and Tables

**Figure 12:**
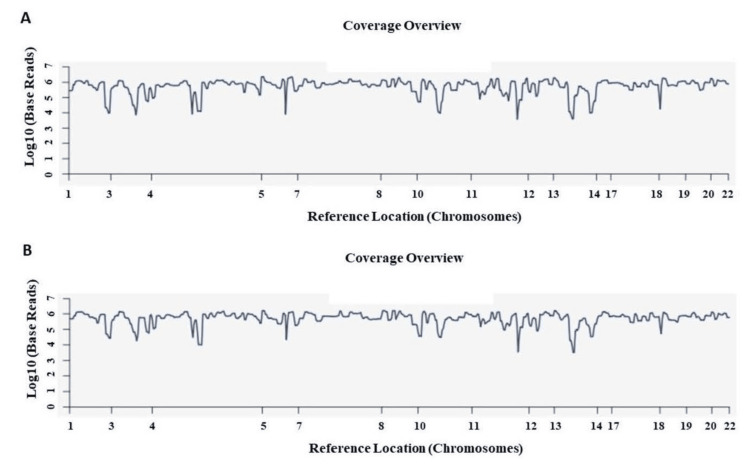
Sequence alignment analysis of the target regions and sample coverage overview Sequence alignment of the target regions (CP.20131001 designed) to the reference genome (Hg 19) and sample coverage analysis was performed by the Ion Torrent Suite software version 5.0.2. CP: cancer panel; Hg: human genome; (A) psammomatous meningioma; (B) angiomatous meningioma

## Appendices

Appendix A 

Appendix B 

**Table 7:** Sequencing coverage analysis report generated by Torrent Suite software v.5.0.2

Amplicon read coverage	Psammomatous	Angiomatous
Number of amplicons	189	189
Percent assigned amplicon reads	95.75%	95.68%
Average reads per amplicon	31,467	35,927
Uniformity of amplicon coverage	89.42%	86.77%
Amplicons with at least 1 read	100%	100%
Amplicons with at least 20 reads	100%	100%
Amplicons with at least 100 reads	100%	100%
Amplicons with at least 500 reads	98.41%	96.30%
Amplicons with no strand bias	97.35%	97.35%
Amplicons reading end-to-end	96.83%	97.35%

Appendix C

**Table 8:** Sequencing coverage analysis report generated by Torrent Suite software v.5.0.2

Target base coverage	Psammomatous	Angiomatous
Bases in target regions	13,311	13,311
Percent base reads on target	84.31%	84.30%
Average base coverage depth	29,855	34,369
Uniformity of base coverage	89.37%	87.22%
Target base coverage at 1x	100%	100%
Target base coverage at 20x	100%	100%
Target base coverage at 100x	100%	100%
Target base coverage at 500x	98.32%	96.33%
Target bases with no strand bias	96.56%	96.63%
Percent end-to-end reads	94.16%	94.26%

Appendix D 

**Table 9:** Quality metrics of missense variants found in the angiomatous meningioma tumour cDNA: coding DNA; INDEL: insertion and deletion; MNV: multi-nucleotide variant; SNV: single nucleotide variant; AA: amino acid; SIFT: sorting intolerant from tolerant

Variant type	Genes	Exon	cDNA changed	AA changed	Variant effect	SIFT	PolyPhen2	p-value	Phred score
SNV	ERBB4	15	c.1737G>T	p. (Lys579Asn)	Missense	0.05	0.911	0.0032	24.9515
SNV	PIK3CA	5	c.955A>T	p. (Asn319Tyr)	Missense		0.965	0.00391	24.0806
SNV	PIK3CA	14	c.2119G>A	p. (Glu707Lys)	Missense		0.998	0.00001	85.7567
SNV	PIK3CA	21	c.3075C>T	p. (Thr1025Thr)	Synonymous			0.00001	809.427
SNV	PDGFRA	15	c.2083A>G, c.2083A>T	p. (Ser695Gly), p. (Ser695Cys)	Missense, missense	0.34, 0.26	0.304, 0.157	0.00001	185.156
SNV	KIT	10	c.1638A>G	p. (Lys546Lys)	Synonymous			0.00001	1020.92
SNV	KIT	13	c.1899A>C	p. (Glu633Asp)	Missense	0	0.998	0.00001	170.859
SNV	KIT	13	c.1901G>A	p. (Arg634Gln)	Missense	0.01	1	0.00001	170.859
SNV	KIT	14	c.2068A>T	p. (Ile690Phe)	Missense	0.09	0.019	0.0032	24.9537
INDEL	KDR	7	c.867delG	p. (Ser290fs)	Frameshift deletion			0.00001	200.073
SNV	KDR	7	c.862T>C	p. (Phe288Leu)	Missense	0.14	0.014	0.00001	318.026
SNV	ABL1	7	c.1143T>C	p. (Asp381Asp)	Synonymous			0.00391	24.0746
SNV	ATM	35	c.5296C>G	p. (Pro1766Ala)	Missense		1	0.00001	61.4032
SNV	PTPN11	3	c.144T>A	p. (Asn48Lys)	Missense	0.18	0.205	0.00001	139.168
INDEL, SNV	CDH1	3	c.297G>T, c.298delG	p. (Leu99Phe), p. (Val100fs)	Missense, frameshift deletion	0.01	0.915	0.00004	43.6949
SNV	SMAD4	12	c.1593G>T	p. (Arg531Arg)	Synonymous			0.00001	1727.24
INDEL	SMARCB1	5	c.521delC	p. (Ala174fs)	Frameshift deletion			0.00001	327.926

Appendix E 

**Table 10:** Quality metrics of missense variants found in the psammomatous meningioma tumour cDNA: coding DNA; INDEL: insertion and deletion; MNV: multi-nucleotide variant; SNV: single nucleotide variant; AA: amino acid; SIFT: sorting intolerant from tolerant

Variant type	Genes	Exon	cDNA changed	AA changed	Variant effect	SIFT	PolyPhen2	p-value	Phred score
SNV	ERBB4	23	c.2842G>A	p. (Asp948Asn)	Missense	0	1	0.00001	68.1889
SNV	PDGFRA	14	c.1921C>G	p. (Leu641Val)	Missense	0	1	0.00257	25.8931
SNV	KDR	11	c.1444T>C	p. (Cys482Arg)	Missense	0.02	1	0.00001	689.766
SNV	KDR	11	c.1416A>T	p. (Gln472His)	Missense	0.03	0.003	0.00001	782.614
INDEL	APC	16	c.3920delT	p. (Ile1307fs)	Frameshift deletion			0.00001	3662.59
SNV, MNV	APC	16	c.3986_3987delACinsGT, c.3987C>T	p. (His1329Arg, p. (His1329His)	Missense, synonymous	0.02	0.09	0.00001	54.511
SNV	ABL1	4	c.799A>C	p. (Thr267Pro)	Missense		0.992	0.0032	24.95
SNV	FGFR2	7	c.872T>G	p. (Ile291Ser)	Missense		0.053	0.00001	585.74
SNV	HNF1A	3	c.544C>T	p. (Gln182Ter)	Nonsense			0.00001	95.8043
SNV	HNF1A	3	c.569C>T	p. (Thr190Ile)	Missense	0.23	0.017	0.00001	87.5267
SNV	HNF1A	3	c.577G>A	p. (Glu193Lys)	Missense	0.01	0.454	0.00023	36.392
SNV	STK11	1	c.209A>G	p. (Glu70Gly)	Missense	0.11	0.568	0.00001	61.4032
SNV	JAK3	13	c.1752C>G	p. (Leu584Leu)	Synonymous			0.00001	61.3864
